# Editorial: New approaches toward understanding challenges in molecular virology: computational techniques and machine learning

**DOI:** 10.3389/fmolb.2024.1472796

**Published:** 2024-09-02

**Authors:** Srirupa Chakraborty, Kun Qu, Jayaraman Valadi

**Affiliations:** ^1^ Department of Chemistry and Chemical Biology, Northeastern University, Boston, MA, United States; ^2^ Department of Biochemistry, Yong Loo Lin School of Medicine, National University of Singapore, Singapore, Singapore; ^3^ Department of Computing and Data Sciences, FLAME University, Pune, Maharashtra, India

**Keywords:** virology, molecular virology, computational virology, machine learning in virology, MD simulation

The ongoing battle between viruses and the host immune system presents numerous challenges that necessitate innovative research approaches. The recent pandemic has starkly highlighted the urgency of understanding viral infection and transmission mechanisms and developing effective therapeutic strategies. Rapid viral mutation, immune evasion, and elusive pathophysiologies underscore the complexity of viral infections. On the other hand, positively harnessing bacteriophages to treat bacterial infections through high specificity and overcoming antibiotic resistance is seeing a big thrust in recent times. Modern computational techniques, particularly those involving machine learning (ML) and molecular dynamics (MD), offer high-throughput, high-resolution, and cost-effective insights, complementing traditional experimental methods. This Research Topic on “New Approaches Toward Understanding Challenges in Molecular Virology” brings together interdisciplinary research aimed at elucidating virology and epidemiology and enhancing our preparedness against future pandemics.

A critical prerequisite for effective computational analyses in virology is the availability of comprehensive and representative databases. The underrepresentation of certain viral genomes, as highlighted in Lee et al., poses significant obstacles. This study demonstrates the stark underrepresentation of these phages in public databases, resulting in biases in computational models and limiting the resurgence of phage therapy. Similarly, Alipour et al. leverages machine learning to address the challenges of taxonomic classification, presenting a novel approach that integrates genomic data and host information. Both studies underscore the necessity of diverse and complete datasets to improve the accuracy and efficacy of computational models, which are pivotal for advancing our understanding of viral diversity and facilitating the development of targeted therapies.

The first study in this Research Topic, by Lee et al., dives deep into the genetic diversity of bacteriophages that infect ESKAPE pathogens. These pathogens are of high priority due to their multidrug-resistant nature. The study’s pangenome analysis reveals extensive sharing of core genes among phages infecting the same host, and a scarcity of unique lytic phages and phage proteins with antimicrobial activities. This comprehensive analysis highlights the urgent need to enrich public databases with diverse phage genomes to ensure more accurate and effective applications of phage therapy and computational virology models.

In the second study by Alipour et al., the authors tackle the complexity of astrovirus classification. Astroviruses are genetically diverse, and their classification has traditionally been based on host species, which has become inadequate with the advent of next-generation sequencing. The study proposes a three-pronged machine learning approach combining supervised and unsupervised learning with host information to classify uncharted astrovirus genomes. This method not only enhances classification accuracy but also identifies potential recombinant sequences, offering a scalable and reliable prediction model for emerging viruses.

Physics-driven approaches, such as molecular dynamics (MD), complement machine learning studies by providing detailed insights into the dynamic behavior of viral proteins, which are critical to their function. The relevant research on noncovalent inhibitors of Valdes-Albuernes et al. employs advanced molecular modeling techniques to account for protein flexibility, thereby enhancing the accuracy of inhibitor predictions. Valdes-Albuernes et al., exemplifies the power of MD simulations in understanding viral protein-ligand interactions. PLpro is a critical target for therapeutic intervention in SARS-CoV-2, and the study’s use of multiple structures of the PLpro binding site to account for protein flexibility provides a more accurate depiction of potential inhibitor interactions. This method, which clusters generated structures to represent the binding site’s flexibility, enhances the predictive power of docking models and can be extended to other viral proteins requiring similar dynamic considerations.

This approach is paralleled by the research (Mohammad et al.), which integrates virtual screening and MD simulations to identify potential therapeutic candidates. Both studies illustrate the importance of considering protein dynamics in drug design and highlight the potential of these methodologies to be applied to other viral targets, broadening their impact beyond SARS-CoV-2 to encompass a wider range of viral pathogens. Here Essa Mohammad et al. integrates multiple computational techniques to identify potential inhibitors of 3CLpro, a key protease in the viral replication. The combination of virtual screening of 7,120 compounds, MD simulations, and pharmacoinformatics profiling led to the identification of four promising inhibitors with high binding affinities and superior docking scores. The methodologies employed in this study can be readily adapted to other viral targets, facilitating the rapid identification and optimization of antiviral drugs.

The integration of diverse computational approaches featured in this Research Topic demonstrates the power of combining *in silico* methods to support experimental research. The use of machine learning for taxonomic classification, molecular dynamics for modeling protein-ligand interactions, and virtual screening for drug discovery collectively contribute to a more comprehensive understanding of viral transmission and evolution. These computational techniques not only accelerate the pace of research but also provide robust frameworks for experimental validation. By bridging the gap between computational predictions and laboratory experiments, these studies pave the way for more effective and targeted therapeutic strategies against viral infections.

The collaborative nature of these studies showcases the importance of interdisciplinary research in tackling the multifaceted challenges and avenues posed by viruses. Research presented in this Research Topic highlights the transformative potential of computational approaches in molecular virology. The integration of machine learning, molecular dynamics, and other computational techniques with traditional experimental methods offers a powerful toolkit for advancing our understanding of viral pathogenesis, improving global virus surveillance techniques, and developing effective vaccines and therapeutic strategies. As we continue to face the threat of emerging and re-emerging viruses, and explore virus-dependent therapeutic strategies, the insights gained from these studies underscore the importance of harnessing computational tools to enhance our preparedness and response to future pandemics. As we move forward, it is imperative to continue expanding and diversifying viral genomic databases, refining computational models, and integrating multidisciplinary approaches. The research efforts seen in this Research Topic serve as a model for future studies, emphasizing the need for ongoing innovation and cooperation in the scientific community and government organizations. By building on the foundations laid by these studies, we can enhance our ability to predict, detect, and mitigate pathogen outbreaks, ultimately leading to a healthier and more resilient global population.

